# Rgnef (p190RhoGEF) Knockout Inhibits RhoA Activity, Focal Adhesion Establishment, and Cell Motility Downstream of Integrins

**DOI:** 10.1371/journal.pone.0037830

**Published:** 2012-05-23

**Authors:** Nichol L. G. Miller, Christine Lawson, Xiao Lei Chen, Ssang-Taek Lim, David D. Schlaepfer

**Affiliations:** Moores Cancer Center, University of California San Diego, La Jolla, California, United States of America; King's College London, United Kingdom

## Abstract

**Background:**

Cell migration is a highly regulated process that involves the formation and turnover of cell-matrix contact sites termed focal adhesions. Rho-family GTPases are molecular switches that regulate actin and focal adhesion dynamics in cells. Guanine nucleotide exchange factors (GEFs) activate Rho-family GTPases. Rgnef (p190RhoGEF) is a ubiquitous 190 kDa GEF implicated in the control of colon carcinoma and fibroblast cell motility.

**Principal Findings:**

*Rgnef* exon 24 floxed mice (Rgnef^flox^) were created and crossed with cytomegalovirus (CMV)-driven Cre recombinase transgenic mice to inactivate Rgnef expression in all tissues during early development. Heterozygous Rgnef^WT/flox^ (Cre+) crosses yielded normal Mendelian ratios at embryonic day 13.5, but Rgnef^flox/flox^ (Cre+) mice numbers at 3 weeks of age were significantly less than expected. Rgnef^flox/flox^ (Cre+) (Rgnef−/−) embryos and primary mouse embryo fibroblasts (MEFs) were isolated and verified to lack Rgnef protein expression. When compared to wildtype (WT) littermate MEFs, loss of Rgnef significantly inhibited haptotaxis migration, wound closure motility, focal adhesion number, and RhoA GTPase activation after fibronectin-integrin stimulation. In WT MEFs, Rgnef activation occurs within 60 minutes upon fibronectin plating of cells associated with RhoA activation. Rgnef−/− MEF phenotypes were rescued by epitope-tagged Rgnef re-expression.

**Conclusions:**

Rgnef−/− MEF phenotypes were due to Rgnef loss and support an essential role for Rgnef in RhoA regulation downstream of integrins in control of cell migration.

## Introduction

Directed cell migration is a physical process that requires regulated changes in cell shape and adhesion to the extracellular matrix (ECM) [1]. Sites of cell adhesion (termed focal adhesions, FAs) are mediated by integrins, transmembrane receptors that couple the ECM to the filamentous actin cytoskeleton [2]. The migration cycle begins with membrane protrusion, FA formation at the cell front, FA linkage to the actin cytoskeleton, the generation of traction and forward cell movement, followed by disassembly of FAs at the cell rear [3]. At FAs, integrins bind ECM proteins such as fibronectin (FN) and multi-protein signaling complexes form in association with integrin cytoplasmic domains that drive the migration cycle in part through regulation of Rho-family GTPase activity [4].

Rho GTPases, including Cdc42, Rac1, RhoA, and RhoC are key effectors of cell migration and actin cytoskeletal dynamics that function as molecular switches cycling between an inactive GDP-bound state and an active GTP-bound form that interacts with downstream targets [5]. Rho GTPases are activated by guanine nucleotide exchange factors (GEFs) that catalyze the exchange of GDP for GTP [6]. Rho GTPases return to an inactive state upon hydrolysis of GTP to GDP, a reaction enhanced by GTPase-activating proteins (GAPs) [7]. Initial steps of integrin binding to FN and cell spreading are associated with transient RhoA inhibition followed by a more prolonged period of RhoA activation associated FA formation and the generation of cell tension [8]. Analysis of knockout fibroblasts revealed the importance of both focal adhesion kinase (FAK) and Src-family tyrosine kinases in promoting signals leading to transient RhoA inhibition downstream of integrins [9], [10]. Integrin-stimulated Src and FAK tyrosine phosphorylation of p190RhoGAP is associated with elevated RhoGAP activity and the transient inhibition of RhoA needed for efficient cell motility-polarity [11], [12], [13], [14]. Our understanding of GEFs involved in facilitating RhoA reactivation and FA formation upon FN adhesion remains incomplete.

There are at least 69 different proteins that comprise an extended GEF family [6], [15]. These GEFs contain a conserved region first identified in a transforming gene from diffuse B-cell-lymphoma (Dbl), designated Dbl-homology (DH) [16], [17]. Many GEFs also contain a pleckstrin homology (PH) domain, known to bind phosphorylated phosphoinositide lipids and promote membrane localization [18]. The GEF DH-PH module is the minimal unit promoting nucleotide exchange, but specificity for Rho-GTPase regulation is mediated by additional targeting interactions unique to different GEF proteins [19].

For integrin signaling, knockdown experiments have identified Lsc/p115RhoGEF, LARG, GEF-H1, and p190RhoGEF (Rgnef) as contributing to RhoA activation, actin stress fiber, and FA formation in response to cell adhesion to FN [20], [21], [22]. LARG and GEF-H1 have been linked to RhoA activation in response to mechanical forces on integrins [23]. Over-expression analyses have revealed partial co-localization of p115RhoGEF, LARG, and Rgnef with integrins at FAs [20], [21]. Rgnef binds directly to FAK through a motif in the Rgnef C-terminal domain, a feature not shared with other GEFs [24]. FAK binding directs Rgnef localization to FAs within fibroblasts and this FAK-Rgnef linkage also functions to promote colon carcinoma motility, invasion, and tumor progression [25]. Thus, FAK associates with both GAPs and GEFs in the spatial and temporal control of RhoA regulation and cell motility [26].

Herein, we present results from the generation of an Rgnef knockout mouse. Loss of Rgnef expression does not prevent embryonic development, but numbers of Rgnef−/− mice obtained at weaning were of smaller size and significantly less in numbers compared to expected Mendelian ratios from Rgnef+/− crosses. Primary Rgnef−/− mouse embryo fibroblasts (MEFs) exhibited reduced haptotaxis migration, wound closure motility, and FA numbers formed on FN. Affinity-binding assays to a nucleotide-free RhoA (G17A) mutant revealed Rgnef activation at 60 min after FN stimulation and Rgnef−/− MEFs exhibited significantly reduced RhoA GTP binding at 60 to 120 min on FN compared to normal Rgnef+/+ MEFs. As Rgnef re-expression rescued Rgnef−/− MEF phenotypic defects, these studies establish the importance of Rgnef in RhoA regulation, FA establishment, and cell migration downstream of integrins.

## Results

### Generation of Rgnef knockout mice

**Figure 1:**
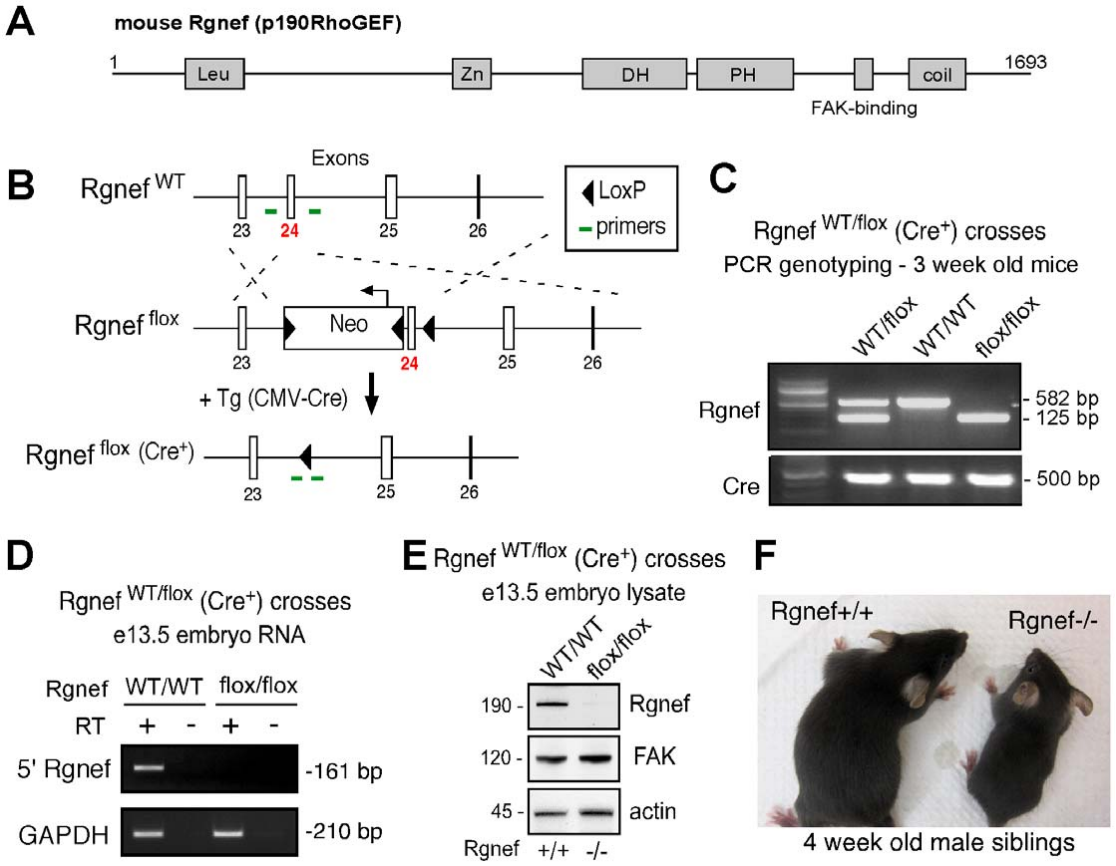
Generation of Rgnef−/− mice. (A) Rgnef schematic. Depicted are the N-terminal leucine-rich (Leu), zinc finger-like (Zn), Dbl- and pleckstrin-homology (DH-PH), FAK binding region, and C-terminal coiled-coil (coil) domains. (B) Genetic targeting strategy to insert LoxP sites and a neomycin selectable marker (Neo) around *Rgnef* exon 24 by homologous recombination (Rgnef ^flox^). Primer binding sites used for PCR screening are indicated (see Table 3). Rgnef ^flox/flox^ mice were crossed with transgenic (Tg) cytomegalorvirus-driven Cre (CMV-Cre) recombinase expressing mice to inactivate Rgnef expression during early development (Rgnef ^flox^, Cre+). (C) Representative PCR genotyping of progeny from Rgnef ^WT/flox^ (Cre+) mouse crosses: Rgnef ^WT/WT^ (582 bp), Rgnef ^WT/flox^ (582 and 125 bp), and Rgnef ^flox/flox^ (125 bp). Cre PCR genotyping (500 bp) is also shown. (D) No truncated Rgnef mRNA transcript detected in Rgnef ^flox/flox^ (Cre+) mice. Embryos were isolated at e13.5 from Rgnef ^WT/flox^ Cre+ mouse crosses, total RNA isolated, and analyzed by RT-PCR using primers 5′ to deleted exon 24 (see Table 3) to Rgnef. GAPDH amplification is a positive control. (E) No Rgnef protein expressed in Rgnef ^flox/flox^ (Cre+) mice. Embryo lysates were immunoblotted with antibodies to Rgnef, FAK, and actin as a control. (F) Representative image of male littermates showing reduced size of Rgnef ^flox/flox^ (Cre+) mice at 4 weeks.

Rgnef, originally termed p190RhoGEF [27], is comprised of N-terminal leucine-rich and cysteine-rich zinc-finger-like regions of unknown function, a central DH-PH domain catalytic region, a FAK interaction motif, and a C-terminal potential coiled-coil region that binds other proteins [28], [29], [30], [31] (Fig. 1A). To determine the necessity of Rgnef expression in mouse development, a transgenic mouse model was created by homologous recombination whereby *Rgnef* exon 24 (coding for part of the DH domain) was flanked by loxP sequences (Rgnef^flox^) (Fig. 1B, Fig. S1). Initial crosses revealed that homozygous Rgnef^flox/flox^ mice developed normally, were fertile, and displayed no detectable phenotype (data not shown).

Rgnef^flox/flox^ mice were then crossed with cytomegalovirus (CMV) Cre transgenic mice (Cre+) [32] to ubiquitously recombine LoxP sites and inactivate Rgnef expression in all tissues during early development and in germ cells (Fig. 1B). Heterozygous Rgnef^WT/flox^ (Cre+) crosses were performed and Cre-driven Rgnef recombination was confirmed by PCR genotyping of tail-extracted DNA from 3 week old littermates (Fig. 1C and Table 1). Reverse-transcriptase (RT) initiated PCR using primers directed 5′ of Rgnef exon 24 (Fig. 1D) or protein immunoblotting (Fig. 1E) did not detect the presence of Rgnef mRNA or protein in Rgnef^flox/flox^ (Cre+) embryos extracted at e13.5 compared to Rgnef^WT/WT^ (Cre+) controls. Although the numbers of Rgnef^WT/WT^ (Cre+), Rgnef^WT/flox^ (Cre+), and Rgnef^flox/flox^ (Cre+) mice analyzed at e13.5 fit normal Mendelian ratios, Rgnef^flox/flox^ (Cre+) mice at 3 weeks of age were significantly less than expected (Table 1) and exhibited an overall smaller body size (Fig. 1F). The cause of this phenotype remains unknown. However, Rgnef^flox/flox^ (Cre+) mice (herein termed Rgnef−/−) grow the same size as Rgnef^WT/WT^ (Cre+) mice (herein termed Rgnef+/+) by 6 to 8 weeks and are fertile. From continued heterozygous matings, decreased Rgnef−/− offspring frequency is still observed when Cre is not present (Rgnef+/+: 31%, n = 13; Rgnef+/−: 49%, n = 20; Rgnef−/−: 20%, n = 8). This result is consistent with a partial lethal perinatal phenotype of Rgnef−/− mice being independent of Cre expression.

**Table 1:** Genotypes of embryos obtained by Rgnef ^WT/flox^ (Cre+) crosses.

	E13.5 (%)	Born (%)
Rgnef WT/WT	10 (21%)	59 (30%)*
Rgnef WT/flox	28 (58%)	104 (53%)
Rgnef flox/flox	10 (21%)	35 (17%)*
Total	48	198

Indicated is the total number of mice obtained at embryonic age 13.5 and born as determined at time of weaning (3 weeks). Pearson's Chi squared analysis (*p<0.05).

**Figure 2:**
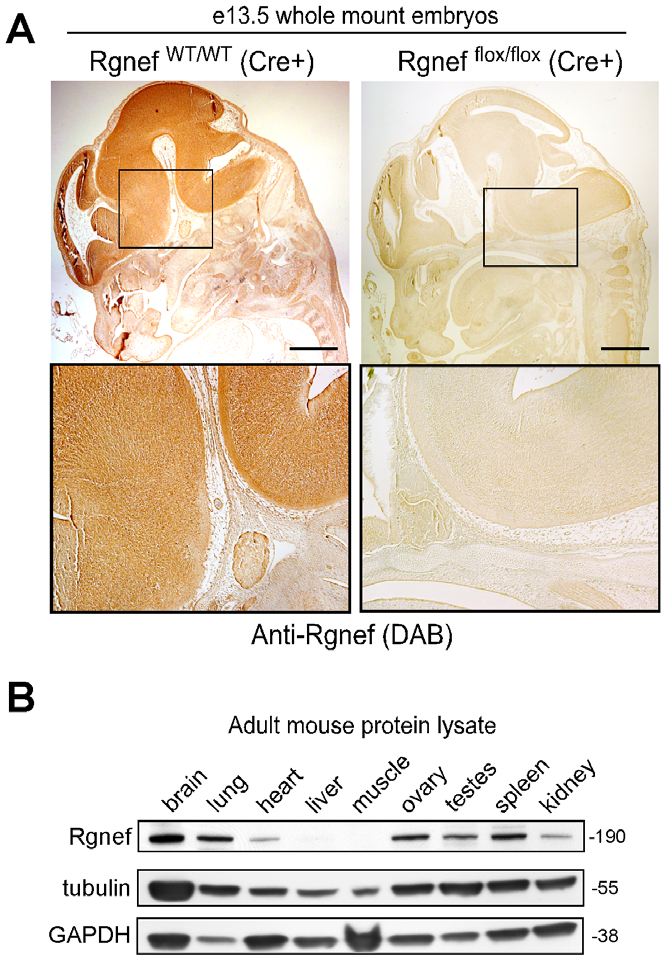
Analyses of Rgnef protein expression. (A) Sagittal sections of e13.5 Cre+ Rgnef ^WT/WT^ Cre+ and Rgnef ^flox/flox^ Cre+ littermate embryos were stained with antibodies to Rgnef, visualized by horseradish peroxidase reactions using diaminobenzidine (DAB, brown). Note loss of DAB staining in Rgnef ^flox/flox^ Cre+ embryos. Scale is 1 mm. (B) The indicated tissues from 10 week old mice were harvested, protein lysates separated by SDS-PAGE, and the same membrane was cut into sections and immunoblotted for Rgnef, α-tubulin and GAPDH.

At e13.5, Rgnef protein expression is maximally detected within the developing brain by immunohistochemical staining with antibodies to Rgnef (Fig. 2A). This is consistent with findings of Rgnef protein expression within mouse brain tissue extracts at e13 and e19 [24]. Immunoblotting analyses of adult mouse tissue revealed the highest relative level of Rgnef protein expression in the brain, lung, ovary, and spleen (Fig. 2B). Low but detectable levels of Rgnef were found in heart, testes, and kidney. Upon over-exposure, an Rgnef band was detected in liver but not in skeletal muscle. These results are consistent with a low ubiquitous pattern of Rgnef expression in most tissues with higher Rgnef levels present in specific organs.

### Rgnef is required for normal MEF cell motility

**Figure 3:**
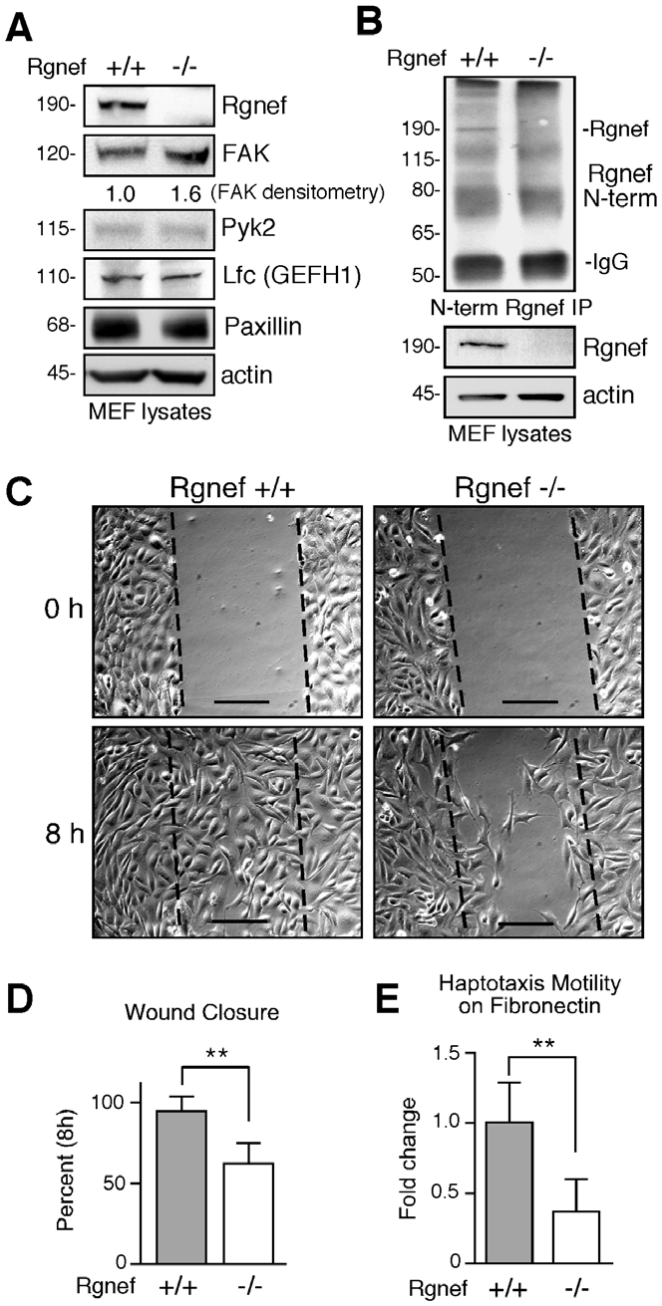
MEF motility requires Rgnef. (A) Protein lysates from primary Rgnef+/+ and Rgnef−/− MEFs were analyzed by immunoblotting for Rgnef, FAK, Pyk2, Lfc (mouse GEFH1 homolog), paxillin, and actin. Densitometry for FAK levels normalized to actin (n = 3 independently-derived cell lines). (B) Anti-Rgnef N-terminal domain antibodies were used for immunoprecipitation and blotting of Rgnef+/+ and Rgnef−/− MEF lysates. Detection of a 190 kDa band in Rgnef+/+ lysates by N-terminal and C-terminal anti-Rgnef antibodies. No specific or potentially truncated (117 kDa) bands were detected in Rgnef−/− MEF lysates. Actin was used as loading control. (C) Phase contrast images (from 0 and 8 h) from a scratch wound migration assay with Rgnef+/+ and Rgnef−/− MEFs. Scale is 100 mm. (D) Wound closure motility was calculated by measuring the distance change between 0 and 8 h in time lapse experiments. Data is the mean percentage ± SD of four independent experiments (** p<0.01). (E) Haptotaxis chamber motility on fibronectin was measured after 3 h. Data is the mean ± SD of three independent experiments normalized to Rgnef+/+ MEFs (** p<0.01).

To assess the necessity of Rgnef in cell function, primary MEFs were isolated from e13.5 Rgnef−/− and Rgnef+/+ embryos (Fig. 3 and Fig. S2). Rgnef mRNA (Fig. S2) and ∼190 kDa Rgnef protein (Fig. 3A) were detected in MEFs from Rgnef+/+ but not Rgnef−/− littermates. Although total FAK levels were slightly increased and verified by densitometry analyses from independently-derived cell lines, no changes were detected in the FAK paralog Pyk2, related GEF-H1/Lfc, or cytoskeletal protein paxillin expression (Fig. 3A). Generation of N-terminal directed polyclonal antibodies to Rgnef did not detect any truncated protein products in Rgnef+/+ or Rgnef−/− MEFs (Fig. 3B). This is consistent with no Rgnef mRNA detected using 5′ end-directed RT-PCR analyses (Fig. 1D). Taken together, these results support the conclusion that floxed deletion of *Rgnef* exon 24 results in the absence of Rgnef expression in embryos and primary MEFs.

Primary MEFs were immortalized via large T antigen expression to facilitate cell culture analyses. Since knockdown experiments MEFs [21] and human colon carcinoma cells [25] support the importance of Rgnef in promoting cell motility, Rgnef−/− and Rgnef+/+ MEFs were analyzed by scratch wound (Figs. 3C and D, Videos S1 and S2) and FN-stimulated haptotaxis transwell (Fig. 3E) migration assays. In both assays, lack of Rgnef expression significantly inhibited cell movement.

### Rgnef facilitates integrin-initiated FA establishment

**Figure 4:**
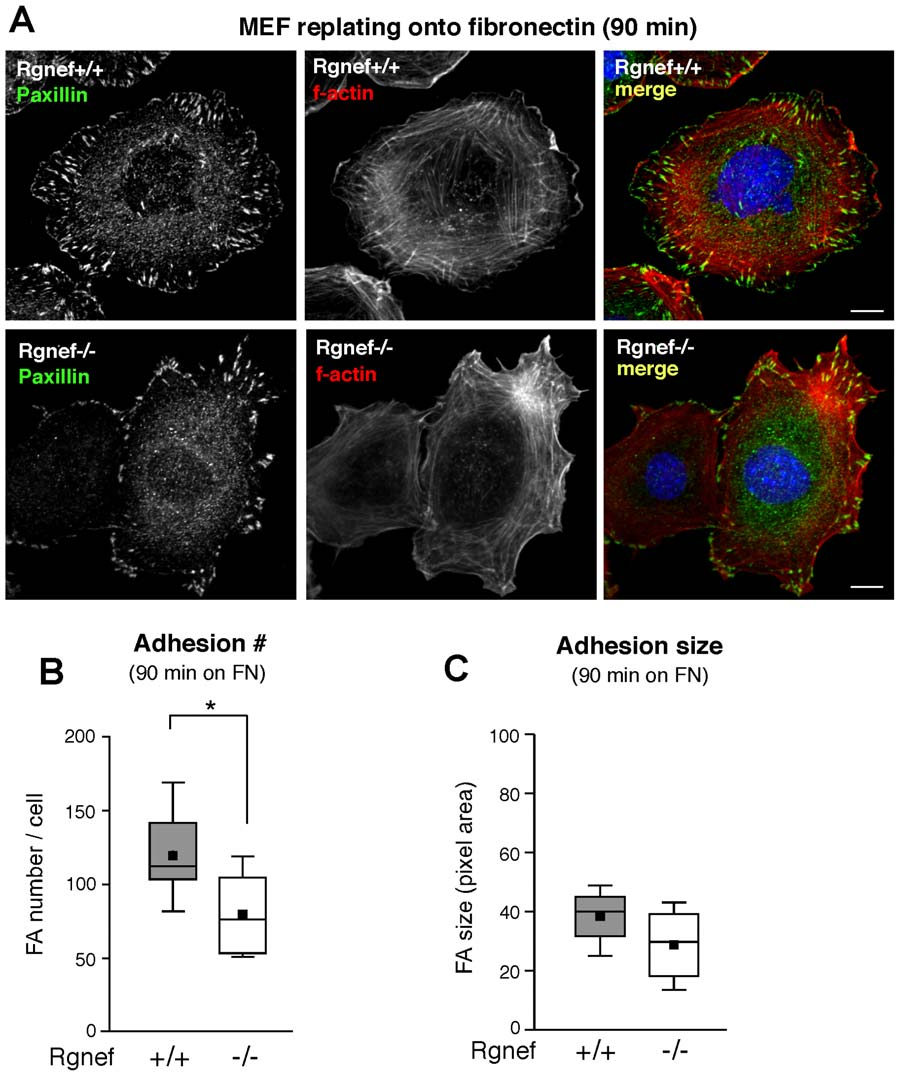
Decreased FA number in Rgnef−/− MEFs on fibronectin (FN) at 90 min. (A) Rgnef +/+ and Rgnef−/− MEFs were plated onto glass coverslips pre-coated with FN (10 µg/ml). Cells were fixed and analyzed for FA formation by paxillin staining (green), f-actin (phalloidin, red), and nuclei were stained with Hoechst (blue) as shown in a merged image. Scale is 10 µm. Significantly-reduced adhesion number (B) and decreased median adhesion size (C) in Rgnef−/− compared to normal (Rgnef+/+) MEFs. Paxillin-stained points were enumerated as FAs by thresholding images using Image J (v1.4) and FA size (pixels) was determined using Cell Profiler (v2.0). Box-and-whisker plots show distribution of the data: mean (*black square*), median (*middle line*), 25^th^ percentile (*bottom line*), 75^th^ percentile (*top line*), and 5^th^ or 95^th^ percentiles (*whiskers*),* p<0.05.

**Figure 5:**
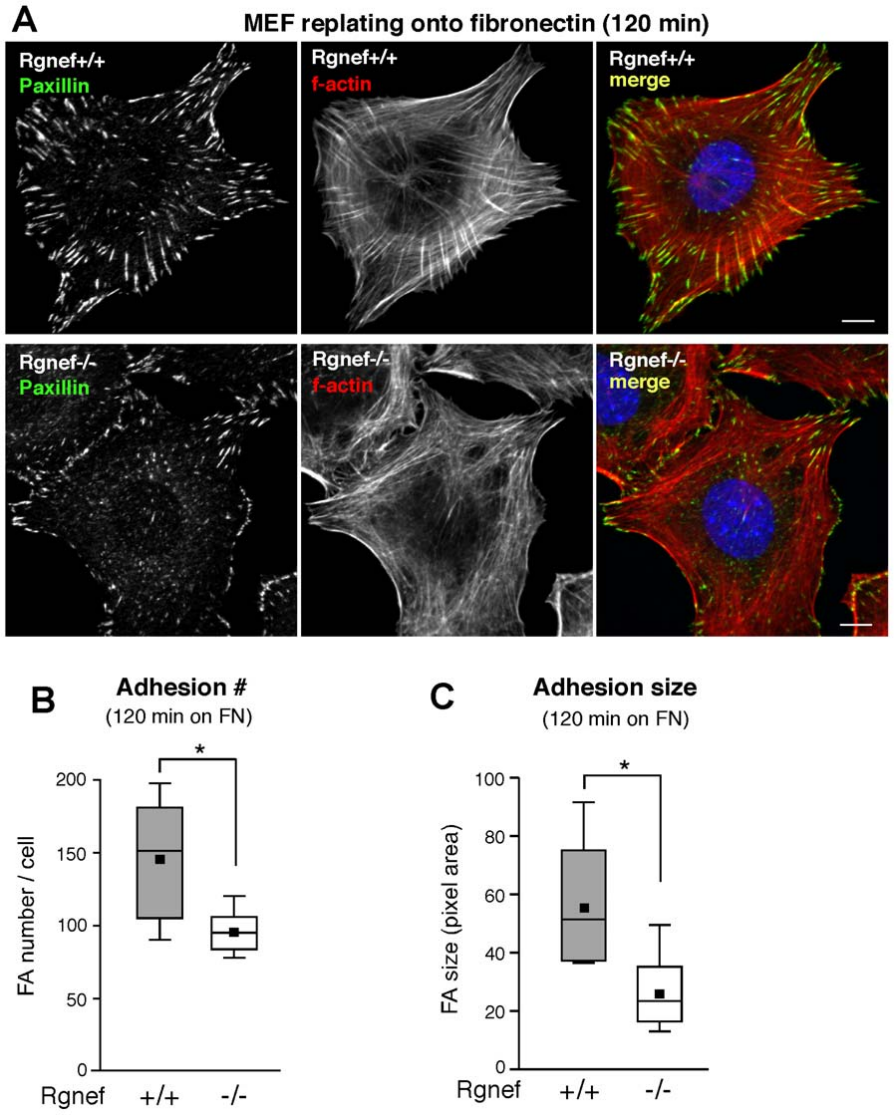
Decreased FA number and size in Rgnef−/− MEFs on fibronectin (FN) at 120 min. (A) Rgnef+/+ and Rgnef−/− MEFs were plated onto glass coverslips pre-coated with FN (10 µg/ml). Cells were fixed and analyzed for FA formation by paxillin staining (green), f-actin (phalloidin, red), and nuclei were stained with Hoechst (blue) as shown in a merged image. Scale is 10 µm. Significantly-reduced adhesion number (B) and size (C) in Rgnef−/− compared to normal (Rgnef+/+) MEFs. Paxillin-stained points were enumerated as FAs by thresholding images using Image J (v1.4) and FA size (pixels) was determined using Cell Profiler (v2.0). Box-and-whisker plots show distribution of the data: mean (*black square*), median (*middle line*), 25^th^ percentile (*bottom line*), 75^th^ percentile (*top line*), and 5^th^ or 95^th^ percentiles (*whiskers*),* p<0.05.

Efficient cell migration requires precise FA assembly-disassembly and stress fiber for tension generation [3], [4]. MEFs will spread and form FAs as detected by paxillin staining with integrated actin stress fibers by 60 to 90 min when replated onto FN-coated surfaces [2]. Rgnef+/+ and Rgnef−/− MEFs were FN replated and analyzed for differences in FA numbers and size at 90 and 120 min (Figs. 4 and 5). Whereas Rgnef−/− MEFs spread equally and exhibited no morphological differences to normal MEFs, loss of Rgnef resulted in significantly fewer FAs at 90 and 120 min (Figs. 4 and 5). Adhesion formation is a dynamic process that can result either in disassembly or maturation corresponding to an increase in FA size [1]. At 90 min on FN, median Rgnef+/+ MEF FA size was 40 pixels compared to Rgnef−/− MEFs at 30 pixels as determined by Cell Profiler software analyses (Fig. 4C). At 120 min on FN, median Rgnef+/+ MEFs FA size increased to 50 pixels whereas Rgnef−/− MEF FA size decreased to 20 pixels (Fig. 5C). The difference in Rgnef+/+ and Rgnef−/− FA size at 120 min was significant and may contribute to the motility defects of Rgnef−/− MEFs. Together, these results support the importance of Rgnef in promoting FA establishment and size after integrin binding to FN.

### Rgnef activation parallels RhoA GTP binding upon FN stimulation

**Figure 6:**
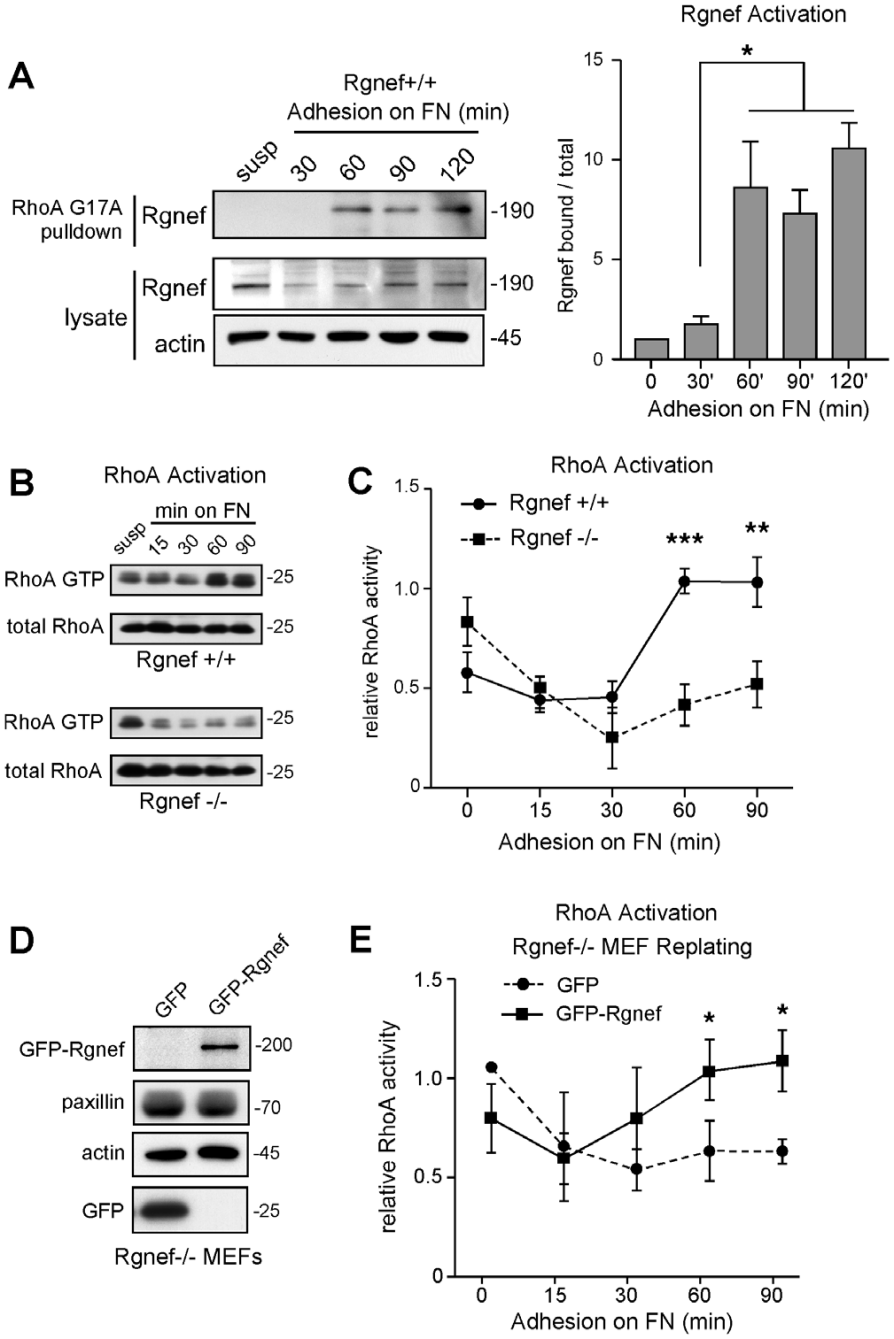
Rgnef activation corresponds with RhoA GTP binding upon FN stimulation. (A) Evaluation of Rgnef activation in lysates of normal MEFs held in suspension for 30 min and then replated onto 10 µg/ml FN for the indicated times. Pull-down assays using GST-RhoA (G17A) and Rgnef immunoblotting reveals binding at 60 to 120 min after FN stimulation (*top*). Total Rgnef and actin levels shown by immunoblotting whole cell lysates (*bottom*). Quantitation of the bound versus total Rgnef by densitometry is shown as the mean ± SD of three independent experiments (* p<0.05, by one-way ANOVA with Tukey post-hoc analysis). (B) Rgnef loss reduces RhoA activation upon FN replating. GTP-bound RhoA was determined in lysates of suspended or FN-stimulated normal (Rgnef+/+) or Rgnef−/− MEFs by GST-Rho binding domain affinity pull-down assays and immunoblotting for RhoA. Whole cell lysate samples were immunoblotted for total RhoA. (C) Quantitation of suspended (0 min) FN-associated RhoA GTP binding by densitometry are means ± SD of three independent experiments expressed relative to total RhoA levels in lysates (** p<0.01, *** p<0.001). (D) Transient expression of GFP or GFP-Rgnef in Rgnef−/− MEFs as detected by GFP or actin immunoblotting or whole cell lysates. Actin and paxillin levels are shown as controls. (E) Rgnef−/− MEFs were transiently-transfected with GFP or GFP-Rgnef, held in suspension for 30 min, and then replated onto 10 µg/ml FN for the indicated times. GTP-bound RhoA was determined by affinity pull-down assays, RhoA immunoblotting, and densitometry. Values are means ± SD of three independent experiments expressed relative to total RhoA levels in lysates (* p<0.05).

Replating experiments with Rgnef+/+ and Rgnef−/− MEFs support the notion that Rgnef activity may influence FA dynamics. Since adhesion to FN triggers the activation of Lsc/p115 and LARG RhoGEFs within MEFs as determined by RhoGEF affinity binding to a nucleotide-free mutant (G17A) of RhoA [20], similar affinity pull-down experiments were performed to evaluate Rgnef activation upon MEF binding to FN (Fig. 6A). Compared to lysates of cells held in suspension or replated on FN for 30 min, Rgnef was significantly activated and bound to GST-RhoA G17A at 60, 90, and 120 min after FN stimulation (Fig. 6A). These results show that Rgnef is activated within 60 min upon cell binding to FN.

Initial MEF binding and spreading on FN is associated with transient RhoA inhibition followed by a more prolonged period of RhoA activation associated FA formation and the generation of cell tension [8]. Analyses of RhoA GTP binding by GST-Rhotekin Rho binding domain affinity pull-down assays revealed increases at 60 and 90 min upon Rgnef+/+ MEF adhesion to FN (Figs. 6B and C). Notably, this parallels the time course of Rgnef activation. Importantly, RhoA GTP binding is significantly inhibited at both 60 and 90 min on FN within Rgnef−/− MEFs (Figs. 6B and C). These results support the importance of Rgnef in facilitating RhoA activation upon FN stimulation.

### Rgnef promotes RhoA activation, increased FA numbers, and cell motility

**Figure 7:**
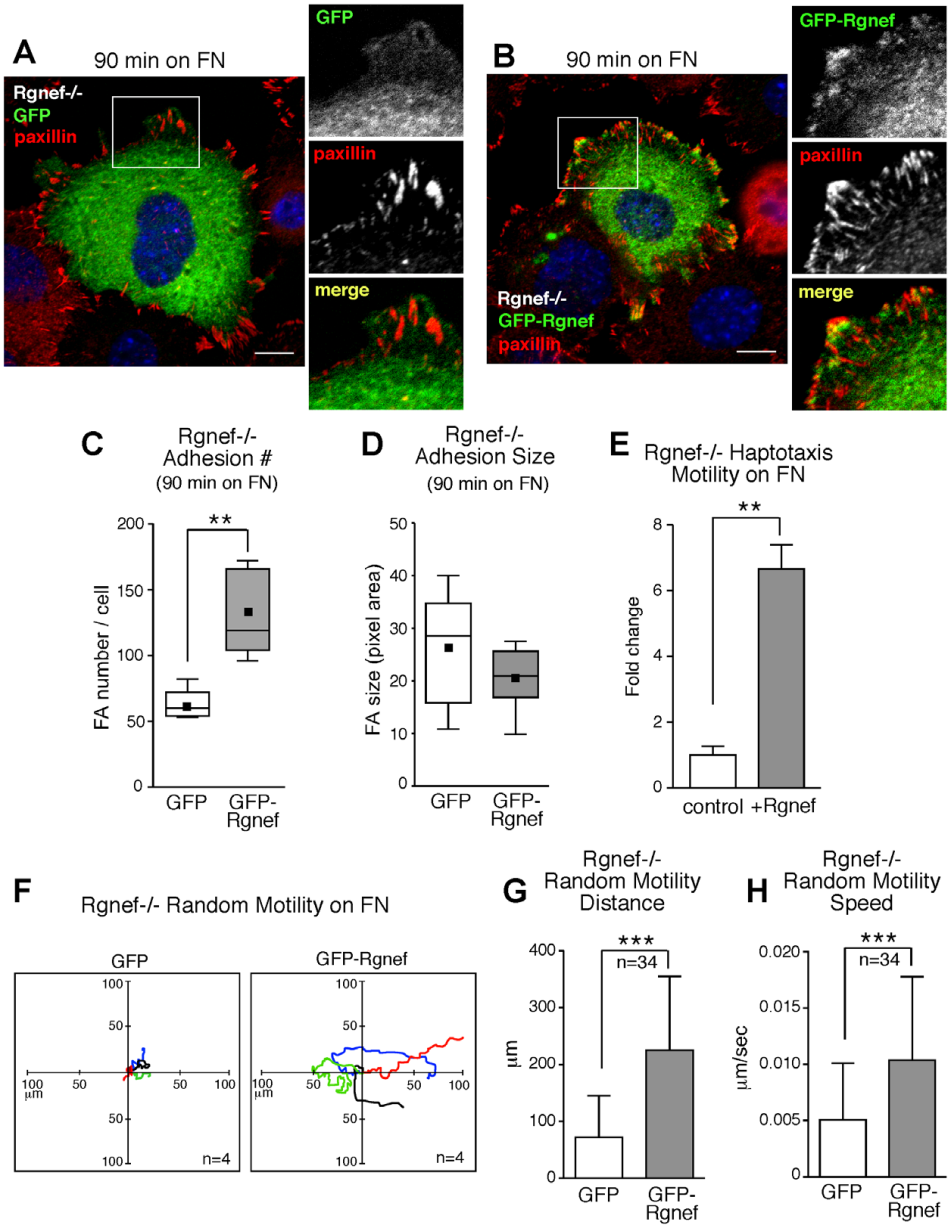
Rgnef re-expression rescues RhoA activation, FA establishment, and motility defects of Rgnef−/− MEFs. (A) GFP control transfected Rgnef−/− MEFs were plated on FN for 90 min, fixed, and analyzed for GFP (green), paxillin (red), and nuclear (DAPI, blue) localization. Scale is 10 µm. Inset, enlarged area of FA staining at periphery of cell (white box). (B) GFP-Rgnef transfected Rgnef−/− MEFs were plated on FN for 90 min, fixed, and analyzed for GFP (green), paxillin (red), and nuclear (DAPI, blue) localization. Scale is 10 µm. Note increased paxillin staining at FAs in GFP-Rgnef-expressing MEFs. Inset, enlarged area of FA staining at periphery of cell (white box). (C) Paxillin-stained points at 90 min on FN were enumerated as FAs by thresholding images using Image J (v1.4) in GFP or GFP-Rgnef transfected Rgnef−/− MEFs (** p<0.01). (D) The size of paxillin-stained points at 90 min on FN enumerated as FAs in C were measured using Cell Profiler (v2.0) in GFP or GFP-Rgnef transfected Rgnef−/− MEFs. (C and D) Box-and-whisker plots show distribution of the data: mean (*black square*), median (*middle line*), 25^th^ percentile (*bottom line*), 75^th^ percentile (*top line*), and 5^th^ or 95^th^ percentiles (*whiskers*). (E) Rgnef−/− MEFs were transfected with plasmids expressing LacZ plus empty vector (control) or LacZ and Rgnef and then analyzed for haptotaxis FN motility (3 h). Motile cells were analyzed for β-galactosidase activity, enumerated, and results are the mean fold change ± SD compared to control transfected cells from four independent experiments (** p<0.01). (F–H) GFP or GFP-Rgnef expressing Rgnef−/− MEFs on 1 ug/ml FN were analyzed by real time microscopy over 5 hours for random motility. (F) Migration paths of 4 representative cells were determined by cell nuclei position and migration of origin was superimposed at 0. Migration distance in µm (G) and overall speed in µm/sec (H) was determined by tracking cells and calculated using Slidebook (v5.0) software (n = 34 per group, values are means ± SD, and p = 0.001).

To determine that Rgnef−/− MEF phenotypes are linked to the loss of Rgnef expression, green fluorescent protein (GFP) or GFP-Rgnef were transiently re-expressed in Rgnef−/− MEFs as a rescue strategy (Fig. 6D). GFP-Rgnef significantly increased RhoA GTP binding at 60 and 90 min after Rgnef−/− MEF replating on FN compared to GFP alone (Fig. 6E). Whereas GFP was primarily distributed in the cytoplasm, a portion of GFP-Rgnef co-localized with paxillin at peripheral FAs when Rgnef−/− MEFs were analyzed by confocal microscopy at 90 min after FN replating (Figs. 7A and B). GFP-Rgnef expression significantly increased the number but not size of FAs formed after 90 min on FN compared to GFP alone (Figs. 7C and D). In parallel, HA-tagged Rgnef re-expression resulted in a 6-fold increase in Rgnef−/− MEF haptotaxis motility on FN after 3 h compared to control-transfected cells (Fig. 7E). When analyzed by real time fluorescent microscopy, GFP-Rgnef increased random Rgnef−/− MEF motility on FN by significantly-increasing the speed and total distance of cell movement compared to GFP-only cells (Figs. 7F–H). Taken together, these results confirm the importance of Rgnef in promoting RhoA activation, FA establishment, and cell motility downstream of integrins.

## Discussion

RhoA GTPase regulation in response to growth factor, G protein linked, and integrin receptor stimuli plays key roles in the formation and regulation of FAs at both leading and trailing edges of migrating cells [4]. Transient knockdown studies have implicated specific signaling pathways connected to the sub-family of RhoA, RhoB, and RhoC GTPases in the control of cell migration [33]. However, results from mouse genetic knockouts (KOs) support the notion of functional overlap within this RhoGTPase sub-family [34]. RhoB KO and RhoC KO mice have no major developmental defects and MEFs derived from these mice exhibit either minor alterations in actin stress fibers (RhoC) or reduced motility and β1 integrin expression (RhoB) [35], [36]. Conditional KO of RhoA in keratinocytes showed that it is not essential for skin development or wound healing in vivo, but RhoA KO keratinocytes exhibit motility defects in culture [37]. In contrast, analyses of RhoA KO MEFs revealed redundant signaling roles with RhoB and RhoC in actomyosin regulation in culture [38]. Thus, understanding the factors of Rho GTPase sub-family regulation is complex and likely associated with the fact that GEFs outnumber Rho GTPases [6]. Moreover, spatiotemporal signal integration within cells is important in the control of cytoskeletal dynamics needed for efficient cell movement [4].

Here we provide the first characterization of an Rgnef KO mouse. From heterozygous crosses, Rgnef−/− mice are found at expected Mendelian ratios at e13.5, but are born at lower than expected Mendelian frequency and exhibit an overall smaller size than Rgnef+/− or Rgnef+/+ littermates. Gross analyses of Rgnef−/− offspring did not reveal apparent abnormalities and this size difference was negligible by 6 to 8 weeks of age. We hypothesize there is an important role for Rgnef in mouse development, but that some type of partial redundancy or compensation may be occurring to lessen or bypass the developmental or physiological restriction point between e13.5 and birth. Although it remains unclear whether the Rgnef KO phenotype parallels RhoA inactivation, embryonic lethal phenotypes are uncommon in other RhoGEF KO mouse models (Table 2). Except for KO of AKAP13 (Brx) that results in heart developmental defects [39] or Trio that results myofibril defects in late embryonic development [40], other RhoGEF KOs have either restricted hematopoietic or other non-lethal phenotypes (Table 2). Despite transient knockdown studies implicating Lsc/p115 RhoGEF and LARG in FN signaling to RhoA [20], mice from these KOs were viable and fertile with either a leukocyte homeostasis defect [41], [42] or smooth muscle hypertension defects that did not necessarily involve integrin signaling connections [43]. Interestingly, as observed with Rgnef KO mice, KOs of the RhoA effector proteins ROCK1 or ROCK2 (Rho-associated protein kinases) also result in partial embryo lethality and birth of small pups [44], [45]. ROCK2 loss was associated with late placental dysfunction and ROCK1 loss with cellular actomyosin bundling defects. Future studies of Rgnef KO embryos in utero between e13.5 and birth will be focused on identifying potential phenotypes as a means to link Rgnef to RhoA signaling in vivo.

**Table 2:** Cytoskeletal-associated RhoA sub-family GEF mouse knockouts.

GEF	Target GTPase	Tissue-Cell Expression	Knockout Phenotype	References
Abr*	RhoA, Rac1, Cdc42	ubiquitous	Viable and fertile, cerebellar and vestibular defects with combined Bcr loss	[49], [50]
Arhgef1/Lsc/p115RhoGEF	RhoA	hematopoietic	Viable and fertile, leukocyte homeostasis defects	[41], [42]
Arhgef5/Tim	RhoA/B, (low RhoC/G)	ubiquitous	Viable and fertile, decrease in dendritic cell migration	[51]
Arhgef12/LARG	RhoA	ubiquitous	Viable and fertile, smooth muscle hypertension defects	[43]
Dbl/Mcf2	RhoA/B/C/G, Rac1, Cdc42	Neural, gonads low level in other tissues	Viable and fertile, dendrite elongation defect	[52]
Lbc/AKAP13/Brx	RhoA	ubiquitous	Early embryonic lethality with heart development defects	[39]
Rgnef/p190RhoGEF	RhoA/C	ubiquitous	Viable and fertile, decreased numbers and size at birth	
Trio	RhoA/G, Rac1	ubiquitous	Embryonic lethal, muscle and neural tissue defects	[40]
Vav1	RhoA/G, Rac1, Cdc42	hematopoietic	Viable and fertile, T cell development defects	[53], [54]
Vav2	RhoA/G, Rac1, Cdc42	ubiquitous	Viable and fertile, B cell defects combined with Vav1 loss	[55]
Vav3	RhoA/G, Rac1, Cdc42	ubiquitous	Viable and fertile, T- and B-cell defects with combined Vav1/2 loss	[56]

* also functions as a GTPase-activating protein.

Many of the restricted hematopoietic or neural defects associated with RhoGEF KOs are associated with alterations in cell movement (Table 2). Surprisingly, there are no reports characterizing cell motility defects RhoGEF KO MEFs in cell culture. It is established that initial MEF binding and spreading on FN is associated with transient RhoA inhibition followed by a more prolonged period of RhoA activation associated FA formation and the generation of cell tension [8], [10]. When compared to wildtype littermate MEFs, Rgnef KO significantly inhibited haptotaxis migration, wound closure motility, FA establishment, and RhoA GTPase activation after FN stimulation. In WT MEFs, Rgnef activation occurs within 60 min upon FN stimulation and this parallels the time course of FN-induced RhoA activation. Importantly, Rgnef KO MEF phenotypes were rescued by Rgnef re-expression; demonstrating that this was a direct result of Rgnef loss. Moreover, our results with Rgnef KO MEFs are in complete agreement with previous studies using short-hairpin RNA interference to knockdown Rgnef expression [21] that resulted in fibroblasts that exhibited motility defects on FN with fewer FAs formed but no alterations in cell spreading.

With regard to the processes involved in FA formation and the control of cell migration, it is important to note that RhoA GTPase regulation is a cycle and that inhibition or constitutive activation of Rho GTPases result in cell migration defects. Too many or too few adhesions formed can prevent efficient cell movement [46]. Moreover, it is overly simplistic to equate decreased FA formation and size differences observed in Rgnef KO MEFs to the inhibition of cell migration. Future studies evaluating the kinetics of FA formation and turnover at both leading and trailing cell projections, studies evaluating the processes of FA maturation or tension generation, and potential differences in adhesome protein content may lead to further mechanistic insights associated with Rgnef KO MEF motility defects. Moreover, Rgnef contains a unique region that binds to FAK and is required for Rgnef localization to FAs [21], [24]. It has been hypothesized that this Rgnef linkage to FAK is key to FN-induced RhoA regulation and future re-expression studies using Rgnef KO MEFs will serve as a powerful system to elucidate the molecular connections of this signaling pathway.

In addition to facilitating RhoA in MEFs, Rgnef expression is elevated as a function of colon cancer tumor grade and stage [25]. A complex of Rgnef, FAK, and paxillin promote colon carcinoma tumor cell motility and matrix degradation in vitro with corresponding increases in tumor growth and surrounding tissue invasion in vivo [25]. Cellular projections that promote matrix degradation are termed invadopodia and Rgnef localizes around these sites to activate RhoC in breast carcinoma cells [47]. Whether Rgnef signaling connections linked to tumor progression involve RhoA or RhoC remain unknown. As Rgnef KO mice are fertile, analyses of these mice crossed to spontaneous mouse tumor models will serve to elucidate the role of Rgnef in promoting tumor invasion and progression.

## Materials and Methods

### Ethics Statement

All procedures were approved by UCSD Institutional Animal Care and Use Committee and mice were maintained in accordance with Association for Assessment and Accreditation of Laboratory Animal Care-approved guidelines.

### Mice

Rgnef exon 24 floxed mice were created by homologous recombination (InGenious Targeting Laboratory, Stony Brook, NY) and the cloning strategy depicted (Fig. 1B). An 8.64 kb region (encompassing Rgnef exons 23–26, Ensembl release 65 - Dec 2011) from a C57BL/6 BAC clone (RPCI23 388E6) was used to construct the targeting vector in pGK-gb2 loxP/FRT Neo. The region was designed such that the short homology arm (SA) extends about 1.42 kb 5′ to exon 24. The long homology arm (LA) ends 3′ to exon 24 and is 6.77 kb long. The loxP flanked Neo cassette is inserted on the 5′ side of exon 24 and the single loxP site is inserted at the 3′ side of exon 24. The target region is 455 bp and includes exon 24 (Fig. S1A). Targeting vector was confirmed by restriction digest and sequencing after each modification. The targeting vector was subcloned into pSP72 prior to linearization (NotI) and electroporation into mouse BA1 (C57BL**/**6 x129**/**SvEv) hybrid embryonic stem (ES) cells. The total size of the targeting construct (including vector backbone and Neo cassette) was 12.84 kb.

After G418 selection, surviving ES clones were expanded for PCR analysis to identify recombinants. Screening primers A1 and A2 were designed upstream of the short homology arm outside the 5′ region used to generate the targeting construct. A list of PCR primers used is provided in Table 3. PCR reactions using A1 or A2 with the UNI primer (located within the Neo cassette) amplify 1.84 and 1.96 kb fragments, respectively. The control PCR reaction was performed using the internal targeting vector primers AT1 and AT2, which are located at the 5′ and 3′ ends, respectively, of the SA. This amplifies a product 1.17 kb in size. Individual clones from positive pooled samples were screened using the A2 and UNI primers. Five positive SA recombinant clones were identified by a 1.8 kb PCR fragment (Fig. S1B) and sequenced for integration using the OUT1 primer. Confirmation of integration of LA region was performed by PCR using LAN1 and Lox1 (Fig. S1B) or SDL2 and Lox1 primers and sequenced using the LAN1 and OUT1 primers to confirm the presence of the third LoxP site. A secondary confirmation of positive clones was performed by Southern Blotting analyses of DNA digested with StuI using a probe targeted against the 3′ LA region (Fig. S1C). Two confirmed clones (252 and 362) were microinjected into C57BL/6 blastocysts. Resulting chimeras with a high percentage agouti coat color were mated to wild-type C57BL/6 mice to generate F1 heterozygous offspring that were genotyped using primers flanking the neomycin cassette insertion site.

**Table 3:** Primers for Rgnef and CMV Cre mouse genotyping and transcript analysis.

Primer	Sequence	Notes
A1	5′-GTAGCCAGCCCTCCCAACAC-3′	forward, short arm of Rgnef flox transgene
A2	5′-GATGGTGGAAGGCATTCTAAGTCC-3′	forward, short arm of Rgnef flox transgene
AT1	5′-GATCCTGTTAGGCTGGCCCTTTC-3′	forward, Rgnef exon 23
AT2	5′-CTGAGCATGCCCACTATCTGCTTTG-3′	reverse, short arm of Rgnef flox transgene
OUT1	5′-GATTAAGTAGAAAGGGCCAGCCTAAC-3′	reverse, Rgnef exon 23
SDL2	5′-CCTGTGAGCAAGCTTTTAAAGCCTC-3′	forward, between exon 24 and 3′ LoxP site
LOX1	5′-GCTCAGGATTCCCCTTTGGC-3′	reverse, long arm of Rgnef flox transgene
LAN1	5′-CCAGAGGCCACTTGTGTGAGC-3′	forward, Neo cassette in Rgnef flox transgene
UNI	5′-AGCGCATCGCCTTCTATCGCCTTC-3′	reverse, Neo cassette in Rgnef flox transgene
PB3	5′-GTAGCCAGCCCTCCCAACAC-3′	forward, southern probe ES cell confirmation
PB4	5′-CTGAGCATGCCCACTATCTGCTTTG-3′	reverse, southern probe ES cell confirmation
RGNEF GENO FWD	5′-ACTGCAGATCAGCATGTCTTG-3′	genotyping, 582 bp wild-type and/or 125 bp recombined flox allele bands
RGNEF GENO REV	5′-GCTGCTATCTCCAAACGCTAT-3′	
CRE FWD	5′-TCGATGCAACGAGTGATGAG-3′	genotyping, 500 bp band in Cre+ mice
CRE REV	5′-TTCGGCTATACGTAACAGGG-3′	
NEO FWD	5′-ACCTTGCTCCTGCCGAGAAAGTAT-3′	genotyping, Rgnef flox transgene, 520 bp band
NEO REV	5′-AGACGTCGCTTGGTCGGTCTTTAT-3′	
RGNEF RT FWD	5′-CTGCAGCCATCACAAAG-3′	RT-PCR, amplifies Rgnef 2957 bp-3141 bp,184 bp band
RGNEF RT REV	5′-ACTGCAGGATCCTTTCCA-3′	
GAPDH RT FWD	5′-ATGGTGAAGGTCGGTGTGAACG-3′	RT-PCR, amplifies GAPDH 51 bp 261 bp, 210 bp band
GAPDH RT REV	5′-GCTTCCCGTTGATGACAAGCT-3′	
RGNEF 5′RT FWD	5′-TGACCAGCCCACGGAACAAATCTA-3′	RT-PCR, amplifies Rgnef 1990 bp-2151 bp, 161 bp band
RGNEF 5′RT REV	5′-AGTTGGCACACTGGAGTGACTCTT-3′	

Rgnef ^flox/flox^ mice were normal, fertile, and were mated to CMV-Cre mice (B6.C-Tg(CMV-cre)1Cgn/J, Jackson Laboratory) to remove the neomycin cassette and Rgnef exon 24. PCR screening for Cre was performed as described [32]. Pregnant females from timed heterozygous Rgnef ^WT/flox^ (Cre+) crosses were euthanized and embryos harvested at embryonic day (e)13.5 for RNA, protein, and primary MEF isolation from Rgnef ^flox/flox^ (Cre+) (Rgnef−/−) and Rgnef ^WT/WT^ (Cre+) (Rgnef+/+) littermates. Genotypes were confirmed by PCR using DNA extracted from embryo tissue.

### Genotyping and Reverse Transcriptase PCR (RT-PCR)

Genotyping conditions were 94°C for 5 minutes, followed by 35 cycles at 94°C for 30 seconds, either 60°C, 58°C, or 56°C (for Neo, Rgnef, or Cre, respectively) for 30 seconds, and 72°C for 1 minute, followed by a 10 minute extension at 72°C.

For RT-PCR, poly-A RNA was isolated using RNeasy (Qiagen) and cDNA was generated using 1 µg RNA and the SuperScript III first-strand synthesis system (Invitrogen). PCR reaction conditions were performed using a BioRad S1000 thermal cycler and GoTaq green master mix (Promega). Primers used are shown in Table 3.

### Reagents and Antibodies

Rabbit polyclonal affinity-purified anti-peptide antibodies to the C-terminal region of Rgnef were created and used as described [25]. Rabbit polyclonal affinity-purified antibodies directed to Rgnef N-terminal domain residues 265–281 (IKLFRKYFWDRAFLVKAC) were generated by 21st Century Biochemicals. Antibodies to β-actin (AC-17) and α-tubulin (DM1A) were from Sigma. Anti-FAK (4.47 clone) was from Millipore. Pyk2 (clone 11) and paxillin (clone M107) antibodies were from BD Biosciences. Antibodies to RhoA (26C4) and GEF-H1/Lfc (Y-20) were from Santa Cruz Biotechnology. Anti-GFP antibodies were from Covance Research. AlexaFluor-488 and AlexaFluor-594 labeled antibodies and Texas Red-phalloidin were from Invitrogen. FN was purified from blood plasma as described [48] or purchased (Sigma).

### Immunohistochemistry

Embryos were fixed in 4% paraformaldehyde (4°C, overnight) and dehydrated in ethanol/water washes prior to paraffin embedding. Sections (10 µm) were floated onto SuperFrost Plus slides (Fisher) and dried overnight at 37°C. Prior to staining, slides were incubated at 60°C for 30 min, deparaffinized in xylene washes, and rehydrated in ethanol/water washes. Antigen retrieval (boiling for 10 min in 10 mM sodium citrate) and peroxidase quenching (0.3% hydrogen peroxide for 10 min) were performed. Sections were incubated in Blocking Buffer (PBS with 5% normal goat serum, 1% BSA, and 0.3% Triton X-100) for 60 min at room temperature and then incubated in anti-Rgnef antibodies (1∶200 in Blocking Buffer) overnight at 4°C. Biotinylated goat-anti-rabbit IgG (1∶300), Vectastain ABC elite, and diaminobenzidine (Vector Labs) were used to visualize Rgnef antibody binding. Images were acquired using an Olympus IX81 microscope (4× objective), an Infinity1 color CCD camera, and whole embryos images stitched together using Adobe Photoshop CS3 software.

### Cells and Constructs

Primary mouse embryo fibroblasts (MEFs) were isolated from e13.5 embryo Rgnef ^flox/flox^ (Cre+) and Rgnef ^WT/WT^ (Cre+) littermates. Briefly, heads were removed for genotyping, tissue was minced with a razor blade, incubated in 0.25% trypsin-EDTA (Gibco) for 5 min at 37°C, and digested with DNase (100 Units/ml). Cells were collected by centrifugation, and grown on 0.1% gelatin-coated culture dishes in Dulbecco's modified Eagle's medium (DMEM) containing 10% fetal bovine serum (FBS), penicillin (100 units/ml), streptomycin (100 µg/ml), MEM non-essential amino acids, sodium pyruvate (1 mM), and ciprofloxacin (20 µg/ml). Primary MEFs were immortalized by retrovirus-mediated large T-antigen (Addgene) expression followed by puromycin selection. Cells were transfected with pEGFP-C1 (Clontech), pEGFP-Rgnef [21], pcDNA3-LacZ (Invitrogen), and pcDNA-HA-Rgnef [24] using JetPrime (Polyplus Transfection, Inc) per the manufacturer's instructions. pCDH-MCS1-GFP and pCDH-MCS1-GFP-Rgnef were used to produce lentivirus as described [21] and Rgnef−/− MEFs were transduced according to standard methods (System Biosciences).

### Biochemical Analyses

For signaling, haptotaxis cell motility or imaging experiments, cells were starved (0.5% serum) 16 h at sub-confluent densities, treated with 0.06% trypsin and 2 mM EDTA in PBS (2.5 min at 37°C), trypsin was inactivated by addition of soybean trypsin inhibitor (0.5 mg/ml) with 0.25% bovine serum albumin (BSA) in DMEM, collected by centrifugation, resuspended in Migration Medium (DMEM with 0.5% BSA), enumerated (ViCell XR, Beckman-Coulter), and held at 37°C (2×10^5^ cells/ml) for 1 h. Acid-washed glass coverslips or plastic culture dishes were coated with FN (10 µg/ml in PBS) overnight, blocked with 1% BSA in PBS for 30 min, and pre-heated to 37°C prior to use. Whole cell lysates were prepared after the indicated times in Extraction Buffer containing 50 mM Hepes, pH 7.4, 150 mM NaCl, 1.5 mM MgCl_2_, 1 mM EGTA, 10 mM sodium pyrophosphate, 100 mM NaF, 10% glycerol, 1% Triton-X-100, 1% SDS, 0.5 mM orthovanadate with phosphatase and protease inhibitors. For immunoprecipitation analyses, lysates were diluted 2-fold in HNTG buffer (50 mM Hepes, pH 7.4, 150 mM NaCl, 0.1% Triton, 10% glycerol), incubated with antibodies (1 µg) for 3 h at 4°C, antibodies collected with Protein A Plus (Millipore) agarose beads, and beads washed at 4°C in 1% Triton-only Extraction Buffer, followed by washes with HNTG buffer, and resolved by SDS-PAGE. GST-Rhotekin Rho binding domain (RBD) and GST-RhoA (G17A) were expressed in bacteria and purified as described [20], [21]. Pull down assays were performed using 20 µg GST-RBD or GST-RhoA G17A bound to glutathione agarose for 60 min at 4°C and then binding determined by RhoA or Rgnef immunoblotting, respectively.

### Cell Migration

#### Haptotaxis

For transient transfection studies, cells were co-transfected with a pCDNA3-LacZ, pCDNA3-Rgnef, or pCDNA empty vector and evaluated after 36 h. MilliCell chambers (8 µm pores; Millipore) were coated on the membrane underside with FN (10 µg/ml) in Migration Medium for 2 h, washed with PBS, and air dried (30 min) prior to use. Cells were collected as described above, 10^5^ cells in 0.3 ml were added to each MilliCell chamber, units were placed into 24-well plates containing 0.4 ml Migration Medium, and incubated for 3 h at 37°C. Cells on the lower membrane surface were fixed and visualized either by crystal violet staining or analyzed for β-galactosidase activity using X-gal as a substrate. Cells per microscopic field were counted (9 fields per chamber) and mean values were obtained from three individual chambers for each experimental point per assay.

#### Wound healing

Glass bottom dishes (MatTek) were coated with FN (1 µg/ml), cells were plated at a subconfluent density (75%), and after 24 h, cells were serum starved overnight, wounded with a pipette tip, washed with PBS, and incubated in growth media containing 0.5 µg/ml of mitomycin-C prior to imaging. Phase contrast images of cells were acquired every 15 min in a humidified 5% CO2 environment at 37°C using an Olympus IX51 microscope, XY-controlled stage with Z focus drive (Olympus), 10× objective (UPLFL, 0.30 NA), and an Orca-ER camera (Hamamatsu) controlled by Slidebook (v5.0) software. Wound closure percentage was calculated by the change in area between 0 and 8 h.

#### Random migration

1×10^4^ cells were seeded onto fibronectin (FN)-coated (1 µg/ml) 6-well plates in growth media for 8 h, then serum-starved overnight. Media was replaced with growth media, phase contrast and GFP fluorescent images were collected at 5-min intervals over 5 h with a 10× lens on an automated stage (Olympus IX51) at 37°C with humidity and CO_2_ regulation. Slidebook (v5.0) software was used to track cell trajectories by nuclear position over time, calculate distance traveled, and average speed of 34 cells per group.

### Immunofluorescence

Cells replated for the indicated time on FN-coated (10 µg/ml) acid-washed glass coverslips (as above) were fixed in 3.7% paraformaldehyde (10 min), permeabilized with 0.1% Triton X-100 in PBS (10 min) and incubated in blocking buffer (2% BSA in PBS) for 1 h. Paxillin antibodies were diluted (1∶300) in blocking buffer and incubated overnight at 4°C. Coverslips were washed in PBS, incubated with AlexaFluor-488 or -594 goat anti-mouse secondary antibodies, Texas Red-Phalloidin, 4′-6-Diamidino-2-phenylindole (DAPI) or Hoechst 33342 (Invitrogen) diluted in blocking buffer (30 min), and mounted using Vectashield (Vector Labs). Images were acquired sequentially using a mercury lamp source, multiband dichroic, single-band exciter, and single band emitter filter sets (Chroma) on dual filter wheels, an Olympus IX81 spinning disc confocal microscope at 60× (PlanApo, N.A. 1.42) and an Orca-AG camera (Hamamatsu) controlled by Slidebook (v5.0) software. Files were cropped, pseudocolored, and adjusted using Adobe Photoshop CS3. Adhesion size (pixels) and number within a cell were determined by analysis of paxillin staining using Cell Profiler (v2.0, Broad Institute) using a pipeline to threshold images and reduce background fluorescent staining in at least 10 cells per group or by using Image J (v1.4).

### Statistical analyses

Data were analyzed using unpaired Students t-test or one-way ANOVA where indicated with GraphPad Prism (5.0 d). Significance was determined at a p value less than 0.05.

## Supporting Information

Figure S1 **Schematic of Rgnef targeting and embryonic stem (ES) cell confirmation.** (A) Shown is the insertion of the neomycin (Neo) cassette 5′ to Rgnef exon 24, loxP sites (triangles), short arm (SA) and long arm (LA) homology regions, 5′ and 3′ genomic regions (dashed lines), and the various primers used for PCR screening of recombinant clones. Primer sequences are listed in Table 3.(B) PCR confirmation of ES clone recombination. A1 and UNI primers were used to amplify a 1.8 kb sequence within the short arm (left). Lox and Lan1 primers were used to amplify a 1.1 kb band within the long arm (right). (C) Southern blot confirmation of ES clone recombination. StuI-digested DNA was electrophoretically-separated on a 0.8% agarose gel, transferred to nylon membrane, and hybridized with a probe generated by primers PB3 and PB4 to give a 19.1 kb band for wild type and a 9.4 kb band for the recombined allele. ES clones 252 and 362 were used for blastocyst injection.
(TIF)

Figure S2 **MEFs were generated from Rgnef+/+ and Rgnef−/− embryos.** (A) Rgnef and Cre genotyping of primary normal (Rgnef+/+) and Rgnef−/− MEFs isolated from e13.5 embryos and established in culture (B) Total RNA isolated from cells from primary Rgnef+/+ and Rgnef−/− MEFs and samples analyzed by RT-PCR using primers to Rgnef and GAPDH.
(TIF)

Video S1 **Rgnef+/+ MEF scratch wound motility.** Primary normal (Rgnef+/+) MEFs were plated at a subconfluent density (75%) on glass bottom dishes (MatTek) coated with FN (1 µg/ml). After 24 h, cells were serum starved overnight, wounded with a pipette tip, washed with PBS, and incubated in growth media containing 0.5 µg/ml of mitomycin-C prior to phase contrast imaging every 15 min in a humidified 5% CO2 environment at 37°C using an Olympus IX51 microscope, XY-controlled stage with Z focus drive (Olympus), 10× objective (UPLFL, 0.30 NA), and an OrcaER camera (Hamamatsu) controlled by Slidebook (v5.0) software. Images were acquired every 15 minutes and assembled into 10 frames per second video spanning 8 h.
(MOV)

Video S2 **Rgnef−/− MEF scratch wound motility.** Primary Rgnef−/− MEFs were imaged as described in in the legend to Video S1.
(MOV)

## References

[1] Gardel ML, Schneider IC, Aratyn-Schaus Y, Waterman CM (2010). Mechanical integration of actin and adhesion dynamics in cell migration.. Annu Rev Cell Dev Biol.

[2] Geiger B, Yamada KM (2011). Molecular architecture and function of matrix adhesions.. Cold Spring Harb Perspect Biol.

[3] Ridley AJ, Schwartz MA, Burridge K, Firtel RA, Ginsberg MH (2003). Cell migration: integrating signals from front to back.. Science.

[4] Parsons JT, Horwitz AR, Schwartz MA (2010). Cell adhesion: integrating cytoskeletal dynamics and cellular tension.. Nat Rev Mol Cell Biol.

[5] Jaffe AB, Hall A (2005). Rho GTPases: biochemistry and biology.. Annu Rev Cell Dev Biol.

[6] Rossman KL, Der CJ, Sondek J (2005). GEF means go: turning on RHO GTPases with guanine nucleotide-exchange factors.. Nat Rev Mol Cell Biol.

[7] Bos JL, Rehmann H, Wittinghofer A (2007). GEFs and GAPs: critical elements in the control of small G proteins.. Cell.

[8] Ren XD, Kiosses WB, Schwartz MA (1999). Regulation of the small GTP-binding protein Rho by cell adhesion and the cytoskeleton.. Embo J.

[9] Ren X, Kiosses WB, Sieg DJ, Otey CA, Schlaepfer DD (2000). Focal adhesion kinase suppresses Rho activity to promote focal adhesion turnover.. J Cell Sci.

[10] Arthur WT, Petch LA, Burridge K (2000). Integrin engagement suppresses RhoA activity via a c-Src-dependent mechanism.. Curr Biol.

[11] Arthur WT, Burridge K (2001). RhoA inactivation by p190RhoGAP regulates cell spreading and migration by promoting membrane protrusion and polarity.. Mol Biol Cell.

[12] Tomar A, Lim ST, Lim Y, Schlaepfer DD (2009). A FAK-p120RasGAP-p190RhoGAP complex regulates polarity in migrating cells.. J Cell Sci.

[13] Holinstat M, Knezevic N, Broman M, Samarel AM, Malik AB (2006). Suppression of RhoA activity by focal adhesion kinase-induced activation of p190RhoGAP: role in regulation of endothelial permeability.. J Biol Chem.

[14] Lim ST, Chen XL, Tomar A, Miller NL, Yoo J (2010). Knock-in mutation reveals an essential role for focal adhesion kinase activity in blood vessel morphogenesis and cell motility-polarity but not cell proliferation.. J Biol Chem.

[15] Schmidt A, Hall A (2002). Guanine nucleotide exchange factors for Rho GTPases: turning on the switch.. Genes Dev.

[16] Eva A, Vecchio G, Rao CD, Tronick SR, Aaronson SA (1988). The predicted DBL oncogene product defines a distinct class of transforming proteins.. Proc Natl Acad Sci USA.

[17] Hart MJ, Eva A, Evans T, Aaronson SA, Cerione RA (1991). Catalysis of guanine nucleotide exchange on the CDC42Hs protein by the dbl oncogene product.. Nature.

[18] Lemmon MA (2008). Membrane recognition by phospholipid-binding domains.. Nat Rev Mol Cell Biol.

[19] Su KC, Takaki T, Petronczki M (2011). Targeting of the RhoGEF Ect2 to the Equatorial Membrane Controls Cleavage Furrow Formation during Cytokinesis.. Dev Cell.

[20] Dubash AD, Wennerberg K, Garcia-Mata R, Menold MM, Arthur WT (2007). A novel role for Lsc/p115 RhoGEF and LARG in regulating RhoA activity downstream of adhesion to fibronectin.. J Cell Sci.

[21] Lim Y, Lim ST, Tomar A, Gardel M, Bernard-Trifilo JA (2008). PyK2 and FAK connections to p190RhoGEF regulate RhoA activity, focal adhesion formation, and cell motility.. J Cell Biol.

[22] Nalbant P, Chang YC, Birkenfeld J, Chang ZF, Bokoch GM (2009). Guanine nucleotide exchange factor-H1 regulates cell migration via localized activation of RhoA at the leading edge.. Mol Biol Cell.

[23] Guilluy C, Swaminathan V, Garcia-Mata R, Timothy O'Brien E, Superfine R (2011). The Rho GEFs LARG and GEF-H1 regulate the mechanical response to force on integrins.. Nature Cell Biol.

[24] Zhai J, Lin H, Nie Z, Wu J, Canete-Soler R (2003). Direct interaction of focal adhesion kinase with p190RhoGEF.. J Biol Chem.

[25] Yu HG, Nam JO, Miller NL, Tanjoni I, Walsh C (2011). p190RhoGEF (Rgnef) promotes colon carcinoma tumor progression via interaction with focal adhesion kinase.. Cancer Res.

[26] Tomar A, Schlaepfer DD (2009). Focal adhesion kinase: switching between GAPs and GEFs in the regulation of cell motility.. Curr Opin Cell Biol.

[27] Gebbink MF, Kranenburg O, Poland M, van Horck FP, Houssa B (1997). Identification of a novel, putative Rho-specific GDP/GTP exchange factor and a RhoA-binding protein: control of neuronal morphology.. J Cell Biol.

[28] van Horck FP, Ahmadian MR, Haeusler LC, Moolenaar WH, Kranenburg O (2001). Characterization of p190RhoGEF, a RhoA-specific guanine nucleotide exchange factor that interacts with microtubules.. J Biol Chem.

[29] Canete-Soler R, Wu J, Zhai J, Shamim M, Schlaepfer WW (2001). p190RhoGEF Binds to a destabilizing element in the 3′ untranslated region of light neurofilament subunit mRNA and alters the stability of the transcript.. J Biol Chem.

[30] Zhai J, Lin H, Shamim M, Schlaepfer WW, Canete-Soler R (2001). Identification of a novel interaction of 14-3-3 with p190RhoGEF.. J Biol Chem.

[31] Meyer D, Liu A, Margolis B (1999). Interaction of c-Jun amino-terminal kinase interacting protein-1 with p190RhoGEF and its localization in differentiated neurons.. J Biol Chem.

[32] Schwenk F, Baron U, Rajewsky K (1995). A cre-transgenic mouse strain for the ubiquitous deletion of loxP-flanked gene segments including deletion in germ cells.. Nucleic Acids Res.

[33] Vega FM, Fruhwirth G, Ng T, Ridley AJ (2011). RhoA and RhoC have distinct roles in migration and invasion by acting through different targets.. J Cell Biol.

[34] Heasman SJ, Ridley AJ (2008). Mammalian Rho GTPases: new insights into their functions from in vivo studies.. Nat Rev Mol Cell Biol.

[35] Liu AX, Rane N, Liu JP, Prendergast GC (2001). RhoB is dispensable for mouse development, but it modifies susceptibility to tumor formation as well as cell adhesion and growth factor signaling in transformed cells.. Mol Cell Biol.

[36] Hakem A, Sanchez-Sweatman O, You-Ten A, Duncan G, Wakeham A (2005). RhoC is dispensable for embryogenesis and tumor initiation but essential for metastasis.. Genes Dev.

[37] Jackson B, Peyrollier K, Pedersen E, Basse A, Karlsson R (2011). RhoA is dispensable for skin development, but crucial for contraction and directed migration of keratinocytes.. Mol Biol Cell.

[38] Melendez J, Stengel K, Zhou X, Chauhan BK, Debidda M (2011). RhoA GTPase Is Dispensable for Actomyosin Regulation but Is Essential for Mitosis in Primary Mouse Embryonic Fibroblasts.. J Biol Chem.

[39] Mayers CM, Wadell J, McLean K, Venere M, Malik M (2010). The Rho guanine nucleotide exchange factor AKAP13 (BRX) is essential for cardiac development in mice.. J Biol Chem.

[40] O'Brien SP, Seipel K, Medley QG, Bronson R, Segal R (2000). Skeletal muscle deformity and neuronal disorder in Trio exchange factor-deficient mouse embryos.. Proc Natl Acad Sci USA.

[41] Francis SA, Shen X, Young JB, Kaul P, Lerner DJ (2006). Rho GEF Lsc is required for normal polarization, migration, and adhesion of formyl-peptide-stimulated neutrophils.. Blood.

[42] Rubtsov A, Strauch P, Digiacomo A, Hu J, Pelanda R (2005). Lsc regulates marginal-zone B cell migration and adhesion and is required for the IgM T-dependent antibody response.. Immunity.

[43] Wirth A, Benyo Z, Lukasova M, Leutgeb B, Wettschureck N (2008). G12-G13-LARG-mediated signaling in vascular smooth muscle is required for salt-induced hypertension.. Nature Med.

[44] Thumkeo D, Keel J, Ishizaki T, Hirose M, Nonomura K (2003). Targeted disruption of the mouse rho-associated kinase 2 gene results in intrauterine growth retardation and fetal death.. Mol Cell Biol.

[45] Shimizu Y, Thumkeo D, Keel J, Ishizaki T, Oshima H (2005). ROCK-I regulates closure of the eyelids and ventral body wall by inducing assembly of actomyosin bundles.. J Cell Biol.

[46] Nobes CD, Hall A (1999). Rho GTPases control polarity, protrusion, and adhesion during cell movement.. The Journal of cell biology.

[47] Bravo-Cordero JJ, Oser M, Chen X, Eddy R, Hodgson L (2011). A Novel Spatiotemporal RhoC Activation Pathway Locally Regulates Cofilin Activity at Invadopodia.. Curr Biol.

[48] Engvall E, Ruoslahti E (1977). Binding of soluble form of fibroblast surface protein, fibronectin, to collagen.. Int J Can.

[49] Kaartinen V, Gonzalez-Gomez I, Voncken JW, Haataja L, Faure E (2001). Abnormal function of astroglia lacking Abr and Bcr RacGAPs.. Development.

[50] Kaartinen V, Nagy A, Gonzalez-Gomez I, Groffen J, Heisterkamp N (2002). Vestibular dysgenesis in mice lacking Abr and Bcr Cdc42/RacGAPs.. Dev Dyn.

[51] Wang D, Grammer JR, Cobbs CS, Stewart JE, Liu Z (2000). p125 focal adhesion kinase promotes malignant astrocytoma cell proliferation in vivo.. J Cell Sci.

[52] Hirsch E, Pozzato M, Vercelli A, Barberis L, Azzolino O (2002). Defective dendrite elongation but normal fertility in mice lacking the Rho-like GTPase activator Dbl.. Mol Cell Biol.

[53] Fischer KD, Zmuldzinas A, Gardner S, Barbacid M, Bernstein A (1995). Defective T-cell receptor signalling and positive selection of Vav-deficient CD4+ CD8+ thymocytes.. Nature.

[54] Tarakhovsky A, Turner M, Schaal S, Mee PJ, Duddy LP (1995). Defective antigen receptor-mediated proliferation of B and T cells in the absence of Vav.. Nature.

[55] Doody GM, Bell SE, Vigorito E, Clayton E, McAdam S (2001). Signal transduction through Vav-2 participates in humoral immune responses and B cell maturation.. Nature Immunol.

[56] Fujikawa K, Miletic AV, Alt FW, Faccio R, Brown T (2003). Vav1/2/3-null mice define an essential role for Vav family proteins in lymphocyte development and activation but a differential requirement in MAPK signaling in T and B cells.. J Exp Med.

