# Supplementary figures and images for: Rgnef (p190RhoGEF) Knockout Inhibits RhoA Activity, Focal Adhesion Establishment, and Cell Motility Downstream of Integrins
Source: PLoS One. 2012 May 23;7(5):e37830. doi: 10.1371/journal.pone.0037830 (PMC3359313; doi:10.1371/journal.pone.0037830)

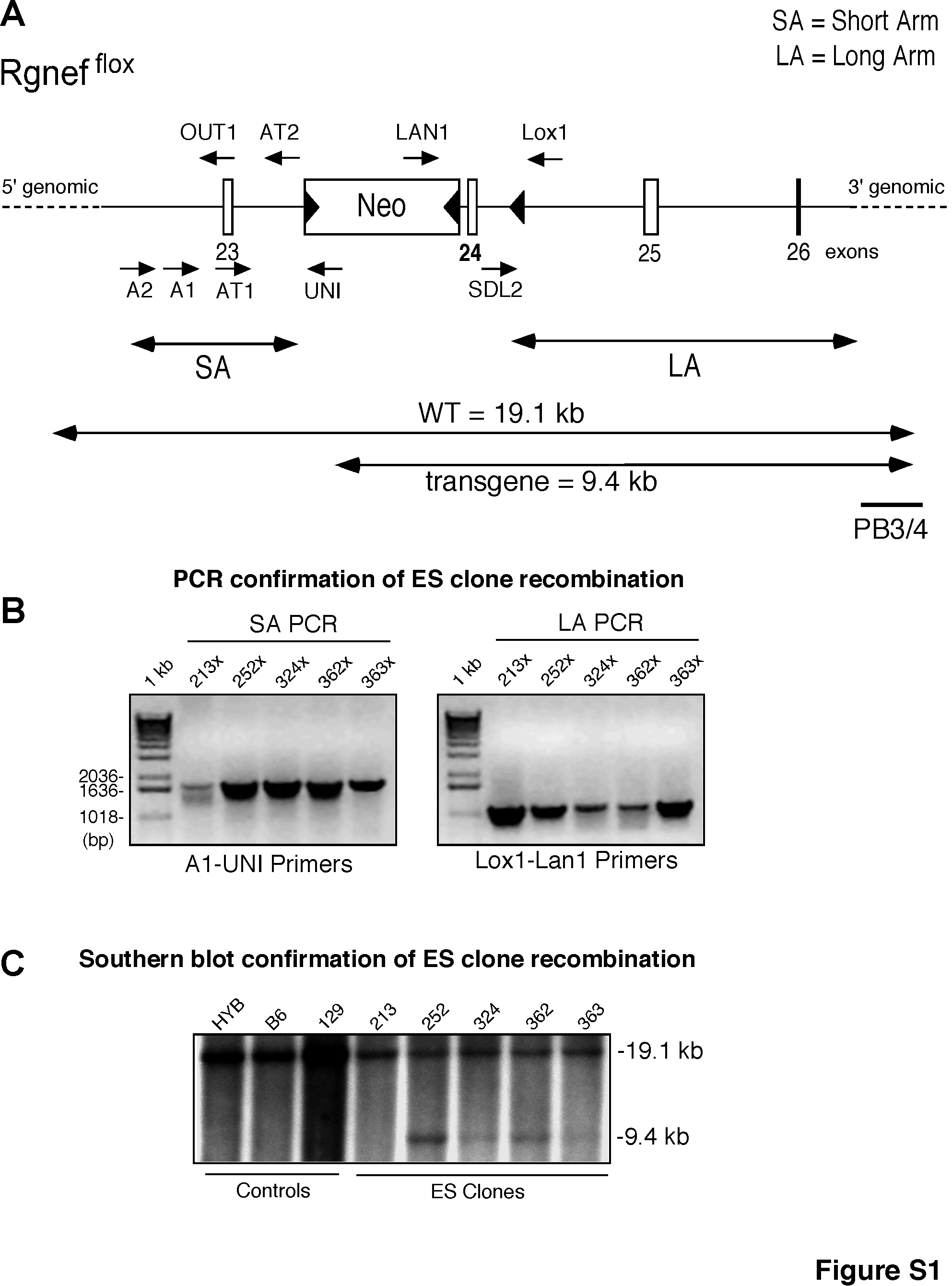

Supplement: Figure S1 — Schematic of Rgnef targeting and embryonic stem (ES) cell confirmation. (A) Shown is the insertion of the neomycin (Neo) cassette 5′ to Rgnef exon 24, loxP sites (triangles), short arm (SA) and long arm (LA) homology regions, 5′ and 3′ genomic regions (dashed lines), and the various primers used for PCR screening of recombinant clones. Primer sequences are listed in Table 3.(B) PCR confirmation of ES clone recombination. A1 and UNI primers were used to amplify a 1.8 kb sequence within the short arm (left). Lox and Lan1 primers were used to amplify a 1.1 kb band within the long arm (right). (C) Southern blot confirmation of ES clone recombination. StuI-digested DNA was electrophoretically-separated on a 0.8% agarose gel, transferred to nylon membrane, and hybridized with a probe generated by primers PB3 and PB4 to give a 19.1 kb band for wild type and a 9.4 kb band for the recombined allele. ES clones 252 and 362 were used for blastocyst injection. (TIF) [file pone.0037830.s001.tif]

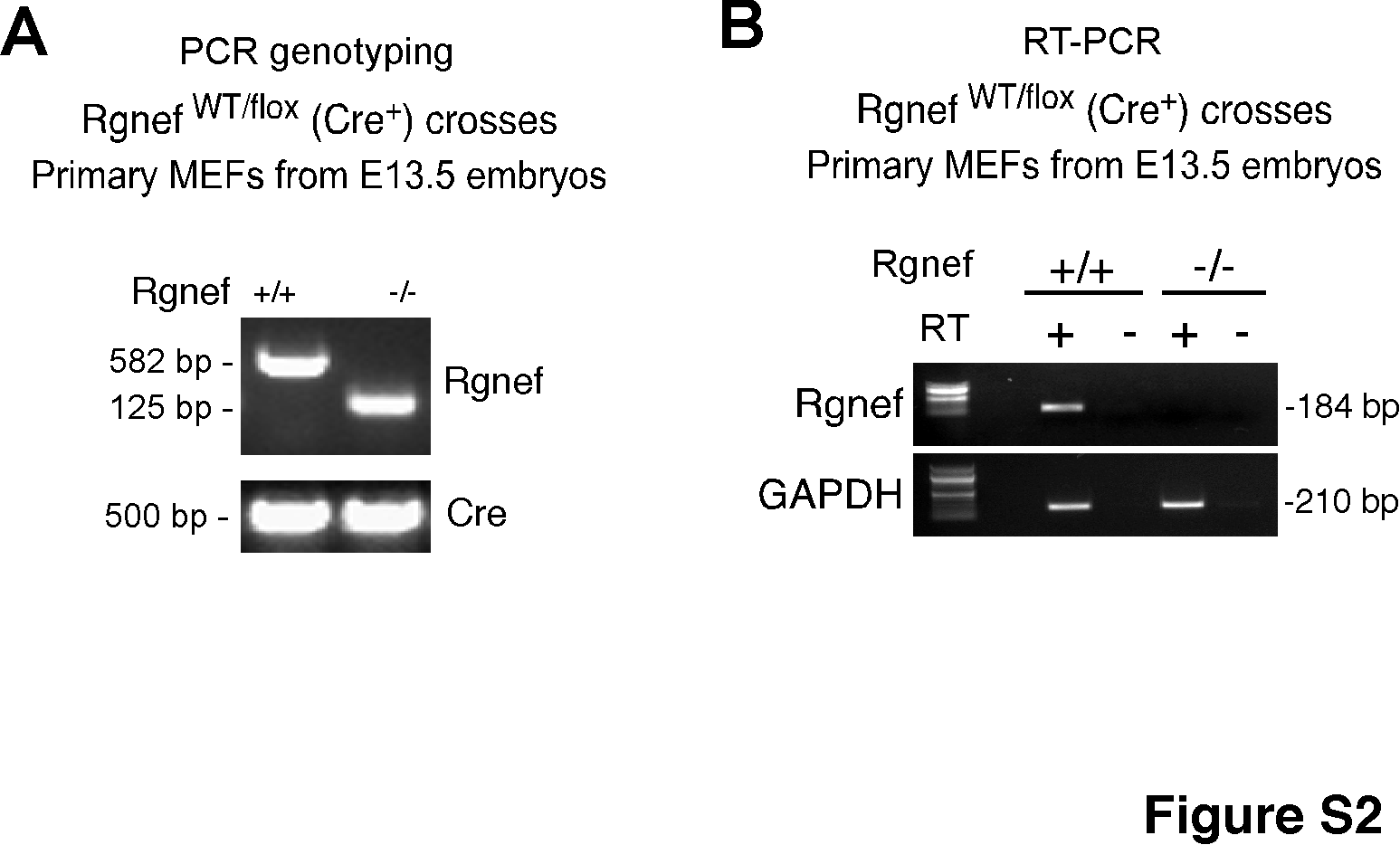

Supplement: Figure S2 — MEFs were generated from Rgnef+/+ and Rgnef−/− embryos. (A) Rgnef and Cre genotyping of primary normal (Rgnef+/+) and Rgnef−/− MEFs isolated from e13.5 embryos and established in culture (B) Total RNA isolated from cells from primary Rgnef+/+ and Rgnef−/− MEFs and samples analyzed by RT-PCR using primers to Rgnef and GAPDH. (TIF) [file pone.0037830.s002.tif]
